# Risk of Cytomegalovirus Viremia Following Transplantation of Hepatitis C‐Viremic Donor Kidneys Into Uninfected Recipients: A Multi‐Center Retrospective Cohort Study

**DOI:** 10.1111/tid.70011

**Published:** 2025-03-06

**Authors:** Vishnu S. Potluri, David Goldberg, Siqi Zhang, Douglas E. Schaubel, Miklos Z. Molnar, Rachel Forbes, Megan E. Sise, James L. Rogers, Vasanthi Balaraman, Anshul Bhalla, David Shaffer, Beatrice P. Concepcion, Raymond T. Chung, Ian A. Strohbehn, Shristi Mapchan, Vikas Vujjini, Akhila Sangadi, Eric Martin, Roy D. Bloom, Alyssa Ammazzalorso, Emily A. Blumberg, Peter P. Reese

**Affiliations:** ^1^ Renal‐Electrolyte and Hypertension Division Hospital of the University of Pennsylvania Philadelphia Pennsylvania USA; ^2^ Center for Clinical Epidemiology and Biostatistics University of Pennsylvania Philadelphia Pennsylvania USA; ^3^ Division of Digestive Health and Liver Disease Department of Medicine University of Miami Miller School of Medicine Miami Florida USA; ^4^ Department of Biostatistics, Epidemiology and Bioinformatics Perelman School of Medicine Philadelphia Pennsylvania USA; ^5^ Department of Internal Medicine Division of Nephrology & Hypertension Spencer Fox Eccles School of Medicine at the University of Utah Salt Lake City Utah USA; ^6^ Department of Surgery Division of Kidney and Pancreas Transplantation Vanderbilt University Medical Center Nashville Tennessee USA; ^7^ Department of Medicine Division of Nephrology Massachusetts General Hospital Boston Massachusetts USA; ^8^ Division of Transplant Surgery University of Tennessee Health Science Center Memphis Tennessee USA; ^9^ Department of Nephrology Kaiser Permanente San Francisco California USA; ^10^ Section of Nephrology Department of Medicine University of Chicago Chicago Illinois USA; ^11^ Department of Medicine Division of Gastroenterology Massachusetts General Hospital Boston Massachusetts USA; ^12^ Nephrology Associates Conway Arkansas USA; ^13^ Division of Infectious Disease Department of Medicine Hospital of the University of Pennsylvania Philadelphia Pennsylvania USA; ^14^ Vanderbilt Center for Transplant Science Vanderbilt University Nashville Tennessee USA

**Keywords:** cytomegalovirus, epidemiology, hepatitis c virus, kidney transplant

## Abstract

**Background:**

Several studies have suggested an increased risk of cytomegalovirus (CMV) viremia among Hepatitis C virus (HCV)‐uninfected recipients of kidney transplants from HCV‐RNA+ deceased donors (HCV D+/R−), but these studies featured small sample sizes and limited ability to address confounding variables.

**Methods:**

We assembled a retrospective cohort of adult kidney transplant recipients at five US centers between 4/1/2015 and 12/31/2020 to determine the association between HCV D+/R− transplants and the outcomes of CMV viremia (> 1000 IU/mL), death‐censored graft failure, and mortality in the first posttransplant year compared to HCV D−/R− transplants. We generated highly similar matched cohorts of HCV D+/R− and HCV D−/R− recipients based on attributes that affect the risk of CMV viremia. We matched exactly on center, CMV donor/recipient serostatus, and antibody induction therapy.

**Results:**

The cohort comprised 275 HCV D+/R− recipients with a mean age of 52.5 years (SD = 10.7); 19% were CMV D+/R−, and 74% received anti‐thymocyte globulin induction. With variable ratio matching, 267 HCV D+/R− recipients were matched to 996 HCV D−/R− recipients. CMV viremia occurred in 15% of HCV D+/R− and 11% of HCV D−R− recipients. In Cox regression, transplantation with an HCV‐RNA+ donor kidney was not associated with a significantly higher risk of CMV viremia (HR 1.3, 95% CI 0.89–1.92) or death‐censored graft loss (HR 0.61, 95% CI 0.31–1.2).

**Conclusion:**

The risk of CMV viremia was not significantly increased among HCV D+/R− kidney recipients. Future studies should examine associations between donor‐derived HCV infection and clinical outcomes of CMV syndrome and disease.

## Graphical abstract

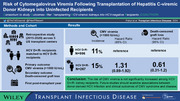

**Abbreviations**
CMV: cytomegalovirus
DAA: direct‐acting antiviral therapy
HCV: hepatitis C virus
HCV D+/R−: HCV‐negative kidney transplant recipient of a kidney from a deceased donor that was HCV‐RNA+
KDRI: Kidney Donor Risk Index
NK: natural killer
OPTN: Organ Procurement and Transplantation Network

## 1 Introduction

Direct‐acting antiviral (DAA) therapy has allowed the transplantation of organs recovered from deceased donors with active HCV infection, defined as detection of HCV‐RNA, into uninfected recipients (HCV D+/R−) and achieved 100% cure rates [1, 2]. Since 2018, over 4500 HCV D+/R− kidney transplants have been performed in the US [3]. Studies have demonstrated that recipients of HCV‐RNA+ kidney transplants have excellent graft survival at 5 years, comparable to transplants from HCV‐uninfected deceased donors [4, 5]. Additionally, the Organ Procurement and Transplantation Network (OPTN) removed the penalty associated with positive results from HCV antibody and/or HCV nucleic acid testing in the Kidney Donor Risk Index (KDRI), such that HCV‐RNA+ kidneys are now rated as having similar quality and projected survival compared to kidneys from HCV‐negative donors [6, 7]. However, gaps in knowledge remain in terms of the potential that donor‐derived HCV infection increases the risk of immunological complications, including opportunistic viral infections [8, 9]. For example, single‐center studies have suggested that HCV D+/R− transplant recipients might experience increased rates of CMV viremia [9].

CMV and HCV can exert diverse immunomodulatory effects on innate, cellular, and humoral domains [10, 11]. This immunomodulation, including long‐term effects on natural killer (NK) cell subpopulations required to control viral replication, have raised the question of whether CMV and HCV co‐infection can affect outcomes in a bidirectional manner [12]. Prior studies provide conflicting evidence about the possibility that donor‐derived HCV infection leads to higher rates of posttransplant CMV infection. For example, in a case series of 53 recipients of HCV D+/R− kidney transplants from Methodist University Hospital, 60% developed CMV viremia, although the median viral load was low: 262 IU/mL [9]. A subsequent translational study examined CMV‐specific T‐cell immunity in a small cohort of kidney recipients and found no association with donor‐derived HCV infection [13]. The multicenter MYTHIC trial reported CMV viremia in 30% of recipients, among whom 17% had CMV viral load > 1000 IU/mL and 13% had probable or definite CMV disease [14]. A single‐center matched cohort study by Shah et al. found higher rates of CMV viremia (median peak viral load 411 IU/mL) associated with transplants from HCV‐RNA+ versus RNA‐negative donors, but the HCV‐RNA+ kidney recipient group had a higher percentage of CMV D+/R− transplants—an important risk factor for CMV viremia. All these studies were limited by small sample sizes [15, 16].

OPTN registry data lack sufficient granularity to examine CMV viremia. Therefore, the Consortium to Study Outcomes After **T**ransplanting HCV‐Viremic Kidneys into HCV‐Negative Recipients (COAUTHOR) consortium was formed to assess infectious and immunological complications involving HCV‐RNA+ organ transplants. We sought to overcome the limitations of prior studies by generating a large, multicenter cohort of highly similar matched pairs of recipients of HCV‐RNA+ versus HCV‐negative kidneys. Our primary aim was to assess the risk of CMV viremia among HCV D+/R− compared to HCV D−/R− recipients within the first posttransplant year.

## 2 Methods

We assembled a retrospective cohort of adults (≥ 18 years at listing) who underwent deceased donor kidney transplant between April 1, 2015, and December 31, 2020 at five centers (University of Pennsylvania, University of Miami, University of Tennessee Health Sciences Center, Massachusetts General Hospital, and Vanderbilt University Medical Center) (Figure S1). We excluded recipients of multiorgan transplants (including kidney–pancreas) and recipients who were HCV‐RNA and/or antibody positive, Hepatitis B virus surface antigen or nucleic acid amplification test positive, and/or seropositive for HIV before transplantation. We excluded recipients of kidneys from donors that were HCV Ab+/HCV‐RNA‐negative.

The institutional review boards approved the study at the five centers. The University of Pennsylvania was the data coordinating center (IRB #833840).

### 2.1 Data Sources

Each center provided baseline data that they had reported to the OPTN on kidney recipients and donors at their respective centers. For the matched pairs, investigators at each site then obtained additional detailed data from the electronic medical record on the initiation of DAA, results of CMV testing, induction immunosuppression, kidney allograft function, and mortality.

### 2.2 Outcomes

The primary binary outcome was the development of CMV polymerase chain reaction (PCR) Quantitation test with a level > 1000 IU/mL within 1 year after transplantation. The rationale for this outcome was that lower concentrations of CMV may not represent meaningful infection but may instead due to highly sensitive CMV assays that detect CMV fragments in the setting of effective immune system control of viral replication [17, 18]. Methods S1 provides additional methods about CMV measurement. Table S1 reports each center's screening strategy for CMV. Secondary outcomes were death‐censored graft loss and mortality.

### 2.3 Primary Exposure

The primary exposure was deceased donor HCV‐RNA status.

### 2.4 Matching Algorithm

To maximize the number of available comparators, we used an optimal “full” matching algorithm, where each HCV D+/R− transplant recipient (“focal patient”) could be matched to a minimum of one to a maximum of five HCV D−/R− deceased donor kidney recipients (“comparator patients”). The matching process was designed to prioritize recipient, donor, and allograft attributes at the time of transplantation that present the greatest risk for CMV infection. Based on published literature and clinical experience, the attributes prioritized were donor/recipient CMV serostatus mismatch, induction therapy, center‐determined protocols for maintenance immunosuppression, and recipient age [19, 20, 21]. We matched exactly on donor/recipient CMV serostatus, induction therapy, and center. Notably, exact matching on the center was also intended to minimize bias in CMV screening and treatment protocols for HCV‐RNA+ and HCV‐negative pairs, which likely have a strong center effect. The algorithm accounted for recipient age, prior transplantation, gender, race, human leukocyte antigen mismatch, cause of kidney disease, calculated panel reactive antibodies at allocation, KDRI at allocation, and cold ischemia time. Additional details about the matching algorithm are available in the Methods S2.

### 2.5 Statistical Analysis

We fit a stratified Cox regression model (R package “coxph”) to compare outcomes between recipients in the focal and comparator groups. We censored patients at 1 year after transplantation or death, whichever was earlier. Outcomes were stratified on the matched set. Because each matched stratum could have a variable number of comparators, we used stratum‐specific averages to generate the overall weighted average distance for each covariate.

## 3 Results

### 3.1 Cohort and Demographic Characteristics

The unmatched cohort comprised 275 HCV D+/R− and 4196 HCV D−/R− kidney transplant recipients at five centers. The mean age of HCV D+/R− recipients was 52.5 years (SD = 10.7). Prior to matching, HCV D+/R− recipients were less likely to be female sex (34% vs. 40%), more likely to be Black race (49% vs. 39%), and more likely to have diabetes as the cause of their kidney disease (43% vs. 26%), respectively, compared to recipients of HCV‐RNA‐negative kidneys. Following optimal matching, 267 out of 275 HCV D+/R− recipients were matched to 996 comparators. The focal (HCV‐RNA D+/R−) and comparator (HCV D−/R−) patients had similar demographic and clinical characteristics with an absolute standardized difference of less than 0.1 (Table 1). All recipients of HCV‐RNA+ kidney transplants treated with DAAs were cured of HCV.

**TABLE 1:** Clinical and demographic characteristics of recipients of kidney transplants from HCV‐RNA+ and HCV‐RNA‐negative deceased donors, pre‐ and post‐match.

	Pre‐match		Post‐match		Post‐match
Variables	HCV RNA D+/R− (*N* = 275)	HCV RNA D−/R− (*N* = 4196)	HCV RNA D+/R− (*N* = 267)	HCV RNA D−/ R− (*N* = 267)^a^	Standardized difference^b^
**Recipient variables**					
Age in years (Mean, SD)	52.5 (10.7)	48.4 (15.0)	52.4 (10.7)	51.3 (10.7)	0.0967
Sex female (%)	93 (33.8%)	1691 (40.3%)	90 (33.7%)	99 (37.1%)	−0.0313
Black race (%)	134 (48.7%)	1636 (39.0%)	131 (49.1%)	148 (55.3%)	−0.0589
**Donor‐recipient CMV serostatus**					
D+/R+ (%)	124 (45.1%)	1892 (45.1%)	123 (46.1%)	123 (46.1%)	0.0000
D+/R− (%)	53 (19.3%)	915 (21.8%)	52 (19.5%)	52 (19.5%)	0.0000
D−/R+ (%)	63 (22.9%)	981 (23.4%)	60 (22.5%)	60 (22.5%)	0.0000
D−/R− (%)	35 (12.7%)	408 (9.7%)	32 (12.0%)	32 (12.0%)	0.0000
**Induction agent**					
Thymoglobulin	203 (73.8%)	3092 (73.7%)	199 (74.5%)	199 (74.5%)	0.0000
Basiliximab	6 (2.2%)	191 (4.5%)	3 (1.12%)	3 (1.12%)	0.0000
Alemtuzumab	66 (24.0%)	913 (21.8%)	65 (24.3%)	65 (24.3%)	0.0000
**Zero HLA mismatch (%)**	4 (1.5%)	190 (4.5%)	0 (0.00%)	0 (0.00%)	0.0000
**History of prior transplant (%)**	16 (5.8%)	452 (10.8%)	15 (5.62%)	14.7 (5.5%)	−0.0031
**Mean PRA (Mean, SD)**	14.0 (27.3)	20.3 (35.4)	12.8 (26.5)	14.8 (28.7)	−0.0647
**Cause of ESKD**					
Diabetes (%)	118 (42.9%)	1090 (26.0%)	114 (42.7%)	90.1 (33.7%)	0.0944
Hypertension (%)	57 (20.7%)	974 (23.2%)	57 (21.3%)	83.9 (31.4%)	−0.0996
Glomerular disease (%)	32 (11.6%)	626 (14.9%)	32 (12.0%)	24.6 (9.2%)	0.0296
Cystic disease (%)	26 (9.5%)	364 (8.7%)	23 (8.6%)	21.6 (8.1%)	0.0013
Other/missing (%)	42 (15.3%)	1142 (27.2%)	41 (13.4%)	46 (17.6%)	−0.0257
**KDRI, mean (SD)**	1.29 (0.24)	1.29 (0.41)	1.30 (0.24)	1.28 (0.29)	0.0504
**CIT in hours, mean (SD)**	19.2 (9.1)	19.3 (12.9)	19.4 (9.01)	18.6 (9.2)	0.0909
**Transplant center**					
Center 1	62 (22.5%)	821 (19.6%)	61 (22.8%)	61 (22.8%)	0.0000
Center 2	16 (5.8%)	334 (8.0%)	14 (5.2%)	14 (5.2%)	0.0000
Center 3	73 (26.5%)	394 (9.4%)	71 (26.6%)	71 (26.6%)	0.0000
Center 4	55 (20.0%)	1571 (37.4%)	54 (20.2%)	54 (20.2%)	0.0000
Center 5	69 (25.1%)	1076 (25.6%)	67 (25.1%)	67 (25.1%)	0.0000

^a^ Each matched comparator transplant recipient received a weight equal to the number of comparators within each matched set. For example, if a matched set has three recipients, then each recipient received a weight of 1/3. The total number of matched comparators was 996, and the total number of weighted comparators was 267.

^b^ Standardized difference accounted for the number of comparators within each matched set.

### 3.2 Primary Outcome of CMV Viremia

**FIGURE 1:**
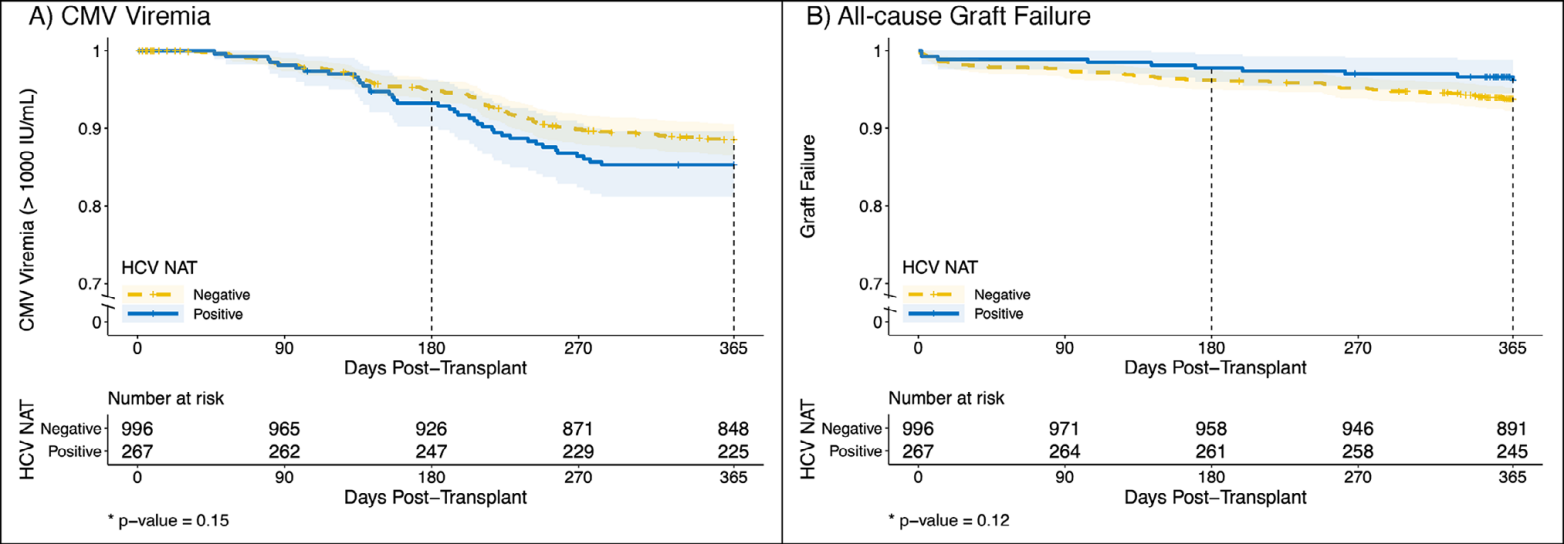
(A) and (B). Kaplan–Meier curve for the outcome of (A) CMV viremia (> 1000 IU/mL) and (B) all‐cause graft failure among recipients of kidney transplants from HCV‐RNA+ and HCV‐negative deceased donors. *p values presented here compare the Kaplan–Meier curves.

Among HCV D+/R− recipients, 91% (range 43%–100%) had at least one CMV PCR quantification test (vs. 77% [range 52%–85%] among HCV D−/R− recipients) (Table S2). The primary outcome of CMV viremia > 1000 IU/mL was detected in 39 (15%) of HCV D+/R− and 29 (11%) of HCV D−/R− recipients in the first year following transplantation. The hazard ratio (HR) for CMV viremia in HCV D+/R− recipients was not significantly higher than in HCV D−/R− recipients (HR 1.31, 95% CI: 0.89–1.92, *p* value = 0.17, Figure 1A). Timing of DAA therapy (< 2 weeks vs. >=( 2 weeks from transplant) was not associated with CMV viremia (HR 0.84, 95% CI: 0.45–1.6, *p* value = 0.6).

CMV D+/R− kidney transplant recipients are considered at high risk for posttransplant CMV complications. We carried out a subgroup analysis which comprised 52 HCV‐RNA+ kidney recipients and matched comparators, all of whom were CMV D+/R−. In the CMV D+/R− subgroup, the risk of CMV viremia was not significantly different between recipients of kidneys from HCV‐RNA+ donors versus HCV‐negative donors (HR 1.11, 95% CI: 0.61–2.00, *p* value = 0.73).

### 3.3 Secondary Outcomes of All‐Cause Graft Loss and Mortality

Transplantation with an HCV‐RNA+ kidney was associated with a lower death‐censored graft loss for HCV D+/R− (HR 0.61 vs. HCV D−/R−, 95% CI: 0.31–1.20, *p* value = 0.153, Figure 1B) and mortality (HR 0.34 vs. HCV D−/R−, 0.1–1.11, *p* value = 0.075); these associations were not statistically significant.

## 4 Discussion

In this multi‐center study of adult kidney transplant recipients, the outcome of CMV viremia was slightly higher among recipients of HCV‐RNA+ versus HCV‐RNA‐negative donor kidneys, but this finding was not statistically significant. This study adds to a growing body of evidence that donor‐derived HCV infection, when treated early after kidney transplantation with DAAs, is unlikely to provoke immunological complications such as viral infections that are difficult to manage [22].

These findings should reassure patients and clinicians about the safety of transplanting organs from HCV‐RNA+ donors and can be placed in the context of related studies. We note that HCV D+/R− recipients underwent more CMV PCR quantification tests than HCV D−/R− recipients, even after matching on donor/recipient CMV serostatus, suggesting that clinicians had concerns about viral coinfections in the setting of donor‐derived HCV. Despite HCV D+/R− recipients undergoing slightly more CMV testing, we did not detect a significantly higher risk of developing CMV viremia > 1000 IU/mL. We acknowledge the possibility that the risk of developing viral coinfections among organ transplant recipients with transient, donor‐derived HCV viremia might be lower than among people with chronic active HCV, where the virus might have a greater opportunity to affect the NK cell subtypes and undermine the response to other latent viruses [10, 12, 23]. Additionally, posttransplant prophylaxis against CMV and early treatment of HCV might also lower the risk of developing CMV viremia among immunosuppressed transplant recipients [24].

This study's methods advance the field. First, we created a multi‐center consortium of large‐volume kidney transplant centers that vary in their immunosuppression protocols, thus increasing the generalizability of the findings. By matching focal and comparator recipients within each center, we limited the potential for bias due to center practices in the surveillance and treatment of CMV and provided a balanced overall estimate of the risk of CMV viremia. Second, rigorous optimal matching algorithms accounted for the most important CMV risk factors. Every HCV‐RNA+ kidney recipient was matched exactly to comparator recipients with the same donor/recipient CMV serostatus and induction treatment. Exact matching on CMV serostatus also enabled precise estimation of the risk of developing CMV viremia within the highest risk subset of recipients (CMV D+/R−). We achieved highly similar distributions of recipient age between groups because older age is associated with susceptibility to infection.

Our study also has limitations. We restricted follow‐up to the first year and might have missed delayed‐onset CMV viremia. However, most episodes of CMV viremia occur during that year and commonly follow the cessation of the CMV prophylaxis [17, 25, 26]. Further, any detrimental effects of HCV infection on immune function would presumably diminish or disappear over the months following HCV cure. A longer follow‐up would also run the risk of confounding by other unrelated posttransplant health events. Second, we matched on the allocation KDRI. The “traditional” KDRI for allocation—in use at the time of study design and data collection—exerted a penalty for donor HCV serostatus. The OTPN has since revised the KDRI and KDPI [6]. Given studies showing that allograft survival is similar between kidneys from donors with and without HCV infection, the KDRI no longer includes HCV [7, 27]. In our study, matching on the traditional KDRI likely explains the slightly lower, but nonsignificant, risk of graft loss and mortality among HCV D+/R− versus HCV D−/R− recipients. However, these small differences in survival should not affect the primary outcome analysis, because we assessed CMV viremia up to 1 year regardless of allograft failure, and deaths were rare. Third, we did not ascertain CMV syndrome or tissue invasive disease. Fourth, it is possible that the study was underpowered to detect a small difference in CMV viremia between the overall matched cohorts as well as in the subgroup of CMV D+/R− transplant recipients. However, from a clinician's perspective, we do not think that a difference on the order of 3%–5% in CMV viremia rates would outweigh the typical benefits of accepting kidneys from HCV‐RNA+ donors, such as shorter waiting times and good graft function.

In summary, transplantation with a kidney from a deceased donor with HCV infection was not significantly associated with CMV viremia. Future studies are required to determine any effect of transient HCV viremia on immunomodulation, such as T‐lymphocyte and NK cell function. Our findings should be confirmed in other cohorts, such as heart transplant recipients.

## Conflicts of Interest

Vishnu Potluri received grants to his institution, from a Monogram grant to the Leonard Davis Institute of Health Economics and the Gift of Life Transplant Foundation. Peter Reese and David Goldberg have received grants to their institution from Merck and Gilead to support trials involving transplants from donors with HCV infection. Peter Reese also received grant support from eGenesis for pre‐clinical studies of xenograft therapies for liver disease, and personal funding from the American Journal of Kidney Diseases for work as an editor. Megan Sise declares research funding from Angion, Otsuka, Gilead, Cabaletta, Novartis, EMD‐Serono, Roche/Genetech, and Merck. She has served on scientific advisory boards or had scientific consulting agreements with Vera, Travere, Calliditas, Mallinckrodt, Novartis, Otsuka, Relay TX, and is a data safety monitoring committee member for Alpine Immune Sciences/Vertex. All other authors have no relevant conflicts to declare.

## Supporting information



Supporting Information

## Data Availability

The data presented in this study will be available from the corresponding author on reasonable request.
