# Supplementary material for: Risk of Cytomegalovirus Viremia Following Transplantation of Hepatitis C‐Viremic Donor Kidneys Into Uninfected Recipients: A Multi‐Center Retrospective Cohort Study
Source: Transpl Infect Dis. 2025 Mar 6;27(3):e70011. doi: 10.1111/tid.70011 (PMC12205285; doi:10.1111/tid.70011)
Supplement: Supplementary file 1 — Supporting Information [file TID-27-e70011-s001.docx]

**Supporting Information**

**Table of Contents**

**Figure S1.** Flowchart depicting cohort generation

**Methods S1.** CMV viremia assessment

**Table S1.** Post-transplant immunosuppression**,** CMV prophylaxis, and CMV monitoring protocols at the study transplant centers

**Methods S2.** Additional details about matching methodology

**Table S2.** Frequency of CMV PCR quantitation testing and CMV-viremia among recipients transplanted with kidneys from HCV-RNA+ (D+/R-) versus HCV- negative (D-/R-) deceased donors

**Figure S1.** Flowchart depicting cohort generation


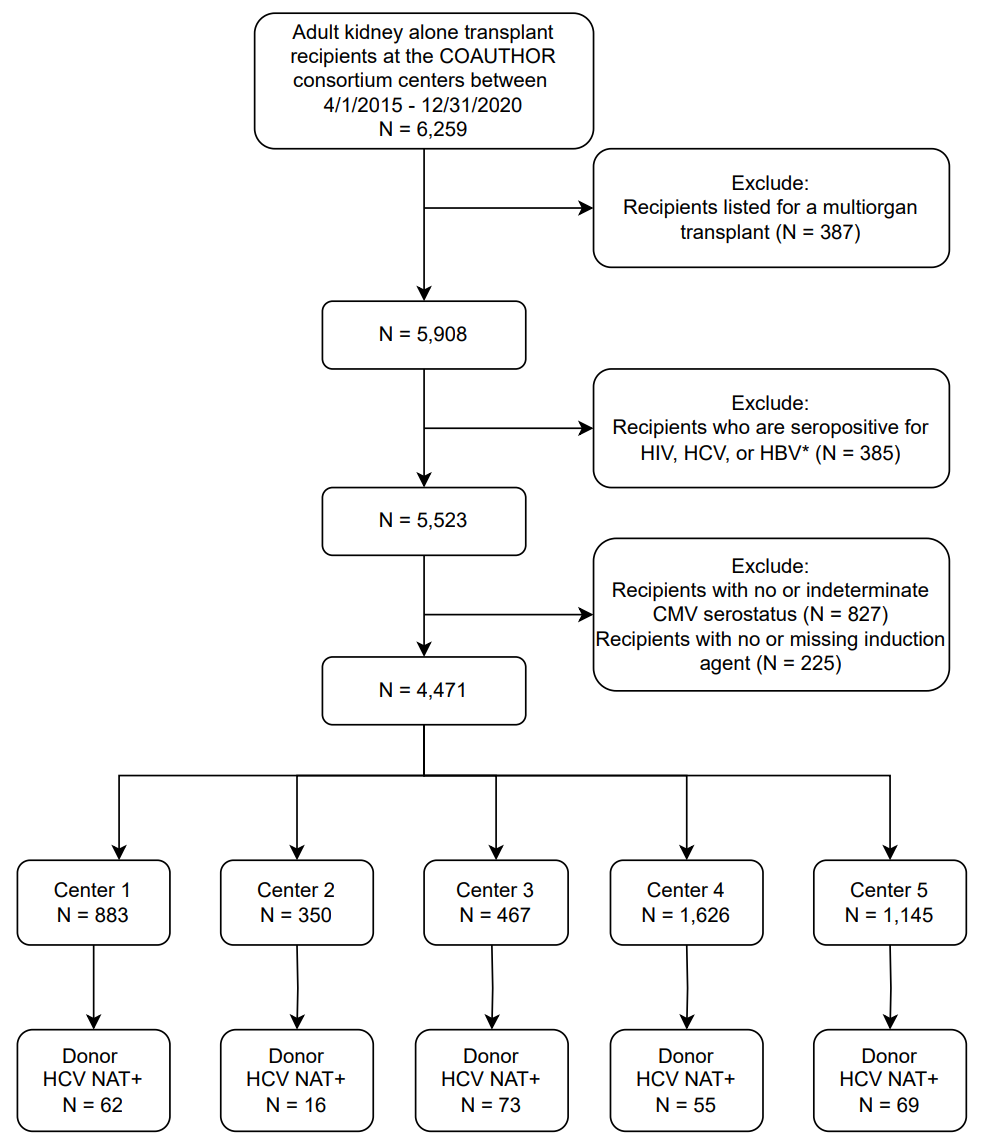


CMV: Cytomegalovirus; HCV: Hepatitis C virus, NAT: Nucleic Acid Amplification Test

* We excluded recipients with either a positive HBV serum antibody level, HBV nucleic acid amplification test, or an HBV surface antigen test.

**Methods S1.** CMV viremia assessment

Investigators at each transplant center reported CMV tests ordered and results. In all five centers, the standard sample for measuring CMV was plasma and the standard approach to CMV quantitation in IU/mL. After reviewing site policies, assays and data reporting in detail, we cannot exclude the possibility that a small number of samples were sent with whole blood, or reported in copies/mL. However, the potential for bias in outcomes is limited because all recipients of kidneys from donors with HCV infection were matched within the same center to recipients of kidneys from HCV-negative donors; therefore, all matched pairs were subject to the same CMV monitoring protocols (see Table S1).

**Table S1.** Post-transplant immunosuppression**,** CMV prophylaxis, and CMV monitoring protocols at the study transplant centers

| **Transplant Center** | **Induction** | **Maintenance Immunosuppression** | **CMV Prophylaxis** | | **CMV Monitoring** | |
| --- | --- | --- | --- | --- | --- | --- |
| **Vanderbilt University Medical Center** | Alemtuzumab and methylprednisolone | High-Immunological Risk Patients (PRA >20%, prior-transplant, ABO or HLA incompatible, history of FSGS, SLE, or MPGN as cause of ESKD): Tacrolimus, MMF, and prednisone.  Low-Immunological Risk Patients: Tacrolimus, MMF, and Solumedrol given on POD 0-2, and no prednisone.  Prednisone tapering protocol for high-risk patients:  POD 0: Solumedrol 500mg  POD 1: Solumedrol 250mg  POD 2: Solumedrol 125mg  POD 3-30: 20mg prednisone  POD 31-45: 15mg prednisone  POD 45-60: 10mg prednisone  POD 61 onwards: 5mg prednisone  Mycophenolate mofetil: 1000mg twice a day | CMV D+/R- | Valganciclovir 450mg daily for 6 months | CMV D+/R- | Post-op months 8, 10, 12 |
|  |  |  | CMV R+ | Valganciclovir 450mg daily for 4 months | CMV R+ | Post-op month 6 |
|  |  |  | CMV  D-/R- | Acyclovir 400mg every 12 hours for 3 months | CMV D-/R- | None |
| **University of Pennsylvania** | Rabbit anti-thymocyte globulin and methylprednisolone | Tacrolimus, MMF, and prednisone  Prednisone tapering protocol:  POD 0: Solumedrol 500mg  POD 1: Solumedrol 250mg  POD 2: Solumedrol 125mg  POD 3: Solumedrol 100mg  POD 4: Prednisone 80mg  POD 5: Prednisone 60mg  POD 6: Prednisone 40mg  POD 7: Prednisone 20mg  POD 8-9: Prednisone 10mg  POD 10 onwards: Prednisone 5mg  Mycophenolate mofetil: 500mg twice a day  Or  Mycophenolate sodium 360mg twice a day | CMV D+/R- | Valganciclovir 900mg daily for 6 months | CMV D+/R- | No standard protocol, but clinicians often check every 2-4 weeks for 1-2 months after completion of prophylaxis |
|  |  |  | CMV R+ | Valganciclovir 450mg daily for 3 months | CMV R+ | None |
|  |  |  | CMV  D-/R- | Valacyclovir 400mg daily for 3 months | CMV D-/R- | None |
| **Massachusetts General Hospital** | Rabbit anti-thymocyte globulin and methylprednisolone | Tacrolimus, MMF, and prednisone.  Prednisone tapering protocol:  Standard:  POD 0: Solumedrol 250mg  POD 1: Solumedrol 125mg  POD 2: Solumedrol 80mg  POD 3: Prednisone 60mg  POD 4: Prednisone 40mg  POD 5-14: Prednisone 20mg  POD 15-21: Prednisone 15mg  POD 22-28: Prednisone 10mg  POD 29 onwards: Prednisone 5mg  Early corticosteroid withdrawal: Discontinue prednisone on day 6 after transplant for low-immunological risk patients.  Mycophenolate sodium: 540mg twice a day | CMV D+/R- | Valganciclovir 900mg daily for 6 months | CMV D+/R- | Consider weekly CMV viral load monitoring for 8-12 weeks after completion of prophylaxis |
|  |  |  | CMV R+ | Valganciclovir 900mg daily for 3 months if CrCl ≥60 mL/min (reduced dose if CrCl <60) | CMV R+ | None |
|  |  |  | CMV  D-/R- | Acyclovir 400mg twice a day for 3 months | CMV D-/R- | None |
| **Jackson Memorial Hospital** | Rabbit anti-thymocyte globulin and methylprednisolone | Tacrolimus, Mycophenolic Acid, and prednisone  Prednisone tapering protocol:  POD 0-2: Solumedrol 500mg  POD 3: Prednisone 80mg (taper at 20mg per day) if high-risk and 20mg if low risk  POD 4: Prednisone 60mg if high risk  POD 5: Prednisone 40mg if high risk  POD 6: Prednisone 20mg if high risk  POD 7: Prednisone 5mg  Low risk: cPRA <40, no DSA, and cross-match negative, not-AA, first transplant, and with immediate graft function  Medium risk: cPRA 40 – 79% and no DSA  High risk: cPRA >79, DSA present, and undergoing desensitization | CMV D+/R- | Valganciclovir 900mg daily for 6 months OR letermovir 480mg daily and valacyclovir 500mg twice a day for 6 months | CMV D+/R- | None, but clinicians monitor frequently |
|  |  |  | CMV R+ | Valganciclovir 900mg daily for 3 months | CMV R+ | None |
|  |  |  | CMV  D-/R- | Valacyclovir 500mg twice a day for 3 months | CMV D-/R- | None |
| **University of Tennessee Methodist University Hospital** | Rabbit anti-thymocyte globulin and methylprednisolone | Tacrolimus, MMF, and prednisone.  Prednisone tapering protocol:  POD 0-2: Solumedrol 500mg  POD 4: 60mg prednisone  POD 5: 40mg prednisone  POD 6: 30mg prednisone  POD 7-14: 20mg prednisone  POD15-21: 15mg prednisone  POD 22-28: 10mg prednisone  POD 29 onwards: 5mg prednisone  Mycophenolate mofetil: 1000mg twice a day  Or  Mycophenolate sodium: 760mg twice a day | CMV D+/R- | Valganciclovir for 6 months | CMV D+/R- | Every 2-4 weeks from months 6-8, and the monthly till month 12 |
|  |  |  | CMV R+ | Valganciclovir for 3 months | CMV R+ | Post-op months 3 to 12 |
|  |  |  | CMV  D-/R- | Valaciclovir for 3 months | CMV D-/R- | Post-op months 3 to 12 |

CMV: Cytomegalovirus, CrCl: Creatinine Clearance, cPRA: calculated Panel reactive antibodies, HLA: Human Leukocyte Antigen, FSGS: Focal Segmental Glomerulosclerosis, SLE: Systemic Lupus Erythematosus, MMF: Mycophenolate mofetil, POD: Post-operative day**Methods S2.** Additional details about matching methodology

Using the donor and recipient covariates described above, we computed a propensity score for treatment assignment (receiving a Hepatitis C virus-RNA+ deceased donor kidney transplant). We then constructed a Mahalanobis distance matrix using recipient age, kidney donor risk index, and calculated panel reactive antibodies. Finally, we used the “optmatch” package in R to build the matched cohort. As described by Rubin and Rosenbaum, covariates were considered balanced if the standardized difference was <0.1.^1,2^ Balance plots used to measure covariate distributions and standardized difference in means were computed using the “cobalt” package in the R statistical software.^3^ Investigators did not assess outcomes until the principal investigators (PR, VSP) confirmed the final matched pairs by assessing standardized differences and covariate distributions.^1,2^

**References**

1. Rosenbaum P. *Observation and experiment.* Harvard University Press; 2018.

2. Rubin DB, Thomas N. Matching using estimated propensity scores: relating theory to practice. *Biometrics.* 1996;52(1):249-264.

3. *cobalt: Covariate Balance Tables and Plots* [computer program]. 2024.

**Table S2.** Frequency of CMV PCR Quantitation testing and CMV-viremia among recipients transplanted with kidneys from HCV-NAT-positive (D+/R-) versus HCV-NAT-negative (D-/R-) deceased donors

|  | HCV D+/R- | HCV D-/R- |
| --- | --- | --- |
|  | Focal patients in the match | Comparators weighted by the number of matched comparators within each matched stratum**^†^** |
| Number of matched recipients (%) |  |  |
| Center 1 | 61 (22.8) | 61 (22.8) |
| Center 2 | 14 (5.2) | 14 (5.2) |
| Center 3 | 71 (26.6) | 71 (26.6) |
| Center 4 | 54 (20.2) | 54 (20.2) |
| Center 5 | 67 (25.1) | 67 (25.1) |
|  |  |  |
| Number of patients that had a CMV PCR Quantitation test performed (%) |  |  |
| Center 1 | 50 (82.0) | 48.8 (80.0) |
| Center 2 | 6 (42.9) | 7.3 (52.4) |
| Center 3 | 71 (100.0) | 53.5 (75.3) |
| Center 4 | 51 (94.4) | 45.8 (84.8) |
| Center 5 | 64 (95.5) | 49.8 (74.3) |
| All | 242 (90.6) | 205.2 (76.8) |
|  |  |  |
| Median number of times CMV PCR Quantitation tests was checked at each center, median (IQR) |  |  |
| Center 1 | 4 (1 - 15) | 0.8 (0.2 - 3.4) |
| Center 2 | 0 (0 - 1.8) | 0.2 (0 - 0.7) |
| Center 3 | 13 (10 - 16.5) | 3 (0 - 5.6) |
| Center 4 | 16 (12 - 19.8) | 2.2 (0.4 - 3.4) |
| Center 5 | 5 (3 - 7) | 0.5 (0.2 - 1) |
| All | 10 (4 - 15) | 1 (0.2 - 3) |
|  |  |  |
| Median number of times CMV was checked – among recipients who had at least one CMV PCR Quantitation test, median (IQR) |  |  |
| Center 1 | 6 (3 - 16) | 1.6 (0.4 - 4.6) |
| Center 2 | 5.5 (1.3 - 9.8) | 0.6 (0.2 - 2.2) |
| Center 3 | 13 (10 - 16.5) | 4.3 (2.8 - 8) |
| Center 4 | 16 (12.5 - 20) | 2.6 (1.4 - 3.6) |
| Center 5 | 5 (3 - 7) | 0.8 (0.4 - 1.2) |
| All | 11 (5 - 16) | 2 (0.6 - 3.6) |
|  |  |  |
| Number of patients with a CMV PCR Quantitation test with > 1,000 IU/mL (%) |  |  |
| Center 1 | 10 (16.4) | 10.3 (16.9) |
| Center 2 | 0 (0.0) | 1.3 (9.2) |
| Center 3 | 12 (16.9) | 4 (5.6) |
| Center 4 | 10 (18.5) | 5 (9.3) |
| Center 5 | 7 (10.4) | 8.4 (12.5) |
| All | 39 (14.6) | 29 (10.9) |

**^†^** We performed optimal matching with a variable number of comparators, where each focal patient could be matched to a minimum of one or a maximum of five comparators. Hence, the outcomes reported here were weighted according to the number of comparators within each matched stratum. For example, if a matched stratum had three comparators, each outcome would receive a weight of 1/3.

HCV: Hepatitis C virus, CMV: Cytomegalovirus, PCR: Polymerase chain reaction
